# A study on efficient detection of network-based IP spoofing DDoS and malware-infected Systems

**DOI:** 10.1186/s40064-016-3569-3

**Published:** 2016-10-26

**Authors:** Jung Woo Seo, Sang Jin Lee

**Affiliations:** Graduate School of Information Security, Korea University, 145 Anam-ro, Seongbuk-gu, Seoul, Korea

## Abstract

Large-scale network environments require effective detection and response methods against DDoS attacks. Depending on the advancement of IT infrastructure such as the server or network equipment, DDoS attack traffic arising from a few malware-infected systems capable of crippling the organization’s internal network has become a significant threat. This study calculates the frequency of network-based packet attributes and analyzes the anomalies of the attributes in order to detect IP-spoofed DDoS attacks. Also, a method is proposed for the effective detection of malware infection systems triggering IP-spoofed DDoS attacks on an edge network. Detection accuracy and performance of the collected real-time traffic on a core network is analyzed thru the use of the proposed algorithm, and a prototype was developed to evaluate the performance of the algorithm. As a result, DDoS attacks on the internal network were detected in real-time and whether or not IP addresses were spoofed was confirmed. Detecting hosts infected by malware in real-time allowed the execution of intrusion responses before stoppage of the internal network caused by large-scale attack traffic.

## Introduction

Hardware performance has seen astonishing advancements with the improving environment of information services. The network infrastructure available today is capable of sending gigabytes of data in just a few seconds. The downside of such developments is that information systems and network infrastructure are more prone to malware-induced cyber-attacks. According a recent report on malware, Linux-based botnets account for the highest proportion of DDoS attacks at 45%, and they pose major threats to the information services of companies when installed in embedded devices such as Wi-Fi, routers, and NAS (Ferguson and Senie 2000; Baker and Savola 2004).

According to a report by Akamai Technologies (Akamai 2015), Linux-based XOR DDoS malware is able to launch DDoS attacks of up to 150 Gbps, and malware-infected systems can attack an average of 20 websites per day. To install and execute malware, attackers acquire the administrative rights of a vulnerable system and run a Shell command in order to install a malware program containing a rootkit function, which allows them to hide their presence.

As demonstrated above, the development of IT has resulted in threats unseen in the past and corporate information service environments are at a greater risk than ever. XOR DDoS malware, one of the most threatening types of malware that can infect Linux-based systems, launches massive IP-spoofed DDoS attacks that paralyze internal networks. Companies have selected Linux-based systems over the more vulnerable Windows operating system as a method of security enforcement, but the various techniques and tools developed by attackers are posing significant threats to businesses.

Past research has focused on the detection of outbound-to-inbound DDoS attacks (Duan et al. 2008; Wang et al. 2007; Feinstein et al. 2003) or the detection of attacks by analyzing information retrieved from botnet agents in internal systems (François et al. 2011; Abu Rajab et al. 2006). However, few studies exist on the detection of network-based attacks when the internal network is under massive volumes of DDoS traffic caused by the IP-spoofed DDoS malware or other infections. For instance, when a DDoS attack occurs due to a malware-infected system in the Demilitarized Zone (DMZ), SYN flooding takes place in the network section from the host system to the security system. The depletion of network resources disrupts network services in the corresponding bandwidth. Rapid detection and response to malware-infected systems is the most effective way of ensuring the availability of the internal network (Perdisci et al. 2013; Beverly et al. 2009; Bremler-Barr and Levy 2005).

This paper applies the DDoS malware finder (DMF) algorithm in order to detect abnormalities when the host of an internal network is infected with DDoS malware based on Spoofed IP Address and launches massive volumes of DDoS attacks. The DMF algorithm derives patterns from feature information of DDoS malware based on Spoofed IP Address and applies the matching rule to enable real-time detection. Malware-infected systems are identified with reference to the DMF table.

The rest of this paper is organized as follows: second section describes the background of the study and related work, third section contains the motivation and challenge, fourth section gives the system overview, fifth section presents the system model, and sixth section is the evaluation. The last section is the conclusion.

## Background and related work

### Background

Malware Must Die team members first detected XOR DDoS in September 2014. A Trojan malware was used to hijack Linux machines in order to build a botnet for DDoS (Akamai 2015).

The bandwidth of DDoS attacks coming from the XOR DDoS botnet has ranged from a few gigabits per second to 150Gbps. The botnet has attacked up to 20 targets per day, 90% of which are in Asia. Two DDoS attacks were caused by the XOR DDoS botnet on the weekend of August 22–23. One of the attacks measured nearly 50 Gbps and the other reached nearly 100 Gbps. XOR DDoS is an example of attackers building botnets from Linux systems instead of Windows-based machines. Other recent examples of Linux-based malware include the Spike DDoS toolkit and IptabLes and IptabLex malware. There is an increasing number of Linux vulnerabilities for malicious actors to target, such as the heap-based buffer overflow vulnerability found earlier this year in the GNU C Library. However, XOR DDoS itself does not exploit a specific vulnerability (Arbor Networks 2015; Akamai 2015).

### Related work

Although a significant amount of literature has been produced on botnet detection, botnet detection approaches using flow analysis techniques have only emerged in the last few years.

Zhao et al. (2013) proposed a new approach to detect botnet activity based on traffic behavior analysis by classifying network traffic behavior using machine learning. Traffic behavior analysis methods do not depend on the packets payload, which means that they can work with encrypted network communication protocols. Network traffic information can usually be easily retrieved from various network devices without affecting significantly network performance or service availability. The proposed approach detects botnet activity by classifying behavior based on time intervals.

BotHunter (John and Tafvelin 2007) consists of intrusion detection system (IDS) components, used to observe inbound and outbound traffic flow, and a dialog correlation engine that generates the flow of bot infections. The two BotHunter plugins are the Statistical sCan Anomaly Detection Engine (SCADE) and the Statistical payLoad Anomaly Detection Engine (SLADE).

SCADE performs inbound scan detection and outbound scan detection by categorizing anomalies as high-severity (HS) or low-severity (LS). SLADE, based on a byte-distribution payload detection technique, inspects the payloads of all packets sent by the service being monitored and provides warnings when the N-gram frequency exceeds the normal range (Gu et al. 2008).

While BotHunter offers a remote repository for users to evaluate bot activities and collect information, it is difficult to detect anomalies in bots that use encrypted channels in order to communicate with the Command and Control (C&C) server and stealth-scanning bots. Since bot infection is determined based on the behavioral patterns of modified bots, bots with signatures updated from variations in traffic patterns are also not easy to identify. To resolve these issues, it is necessary for the server to automatically collect and analyze patterns, as well as to constantly renew them (Tegeler et al. 2012; Gu et al. 2008).

Zeidanloo et al. (2010) proposed a botnet detection approach based on the monitoring of network traffic characteristics in a similar way to BotMiner. In their work, a three stages process of filtering, malicious activity detection and traffic monitoring is used to group bots by their group behavior. The proposed approach divides the concept of flows into time periods of 6 h and clusters these flow intervals with known malicious activity. The effects of different flow interval durations were not presented, and the accuracy of the approach is unknown (Zhao et al. 2013).

Wang et al. (2009) presented a detection approach of peer-to-peer based botnets by observing the stability of control flows in initial time intervals of 10 min. They developed an algorithm which measures the stability of flows and exploits the property that bots exhibit similar behavior in their command search and perform these tasks independently of each other and frequently. They show that by varying parameters in their algorithm, they were able to classify 98% of Storm C&C data as stable, though a large percentage of non-malicious peer-to-peer traffic were also classified as such (Zhao et al. 2013).

## Motivation and challenge

**Fig. 1:**
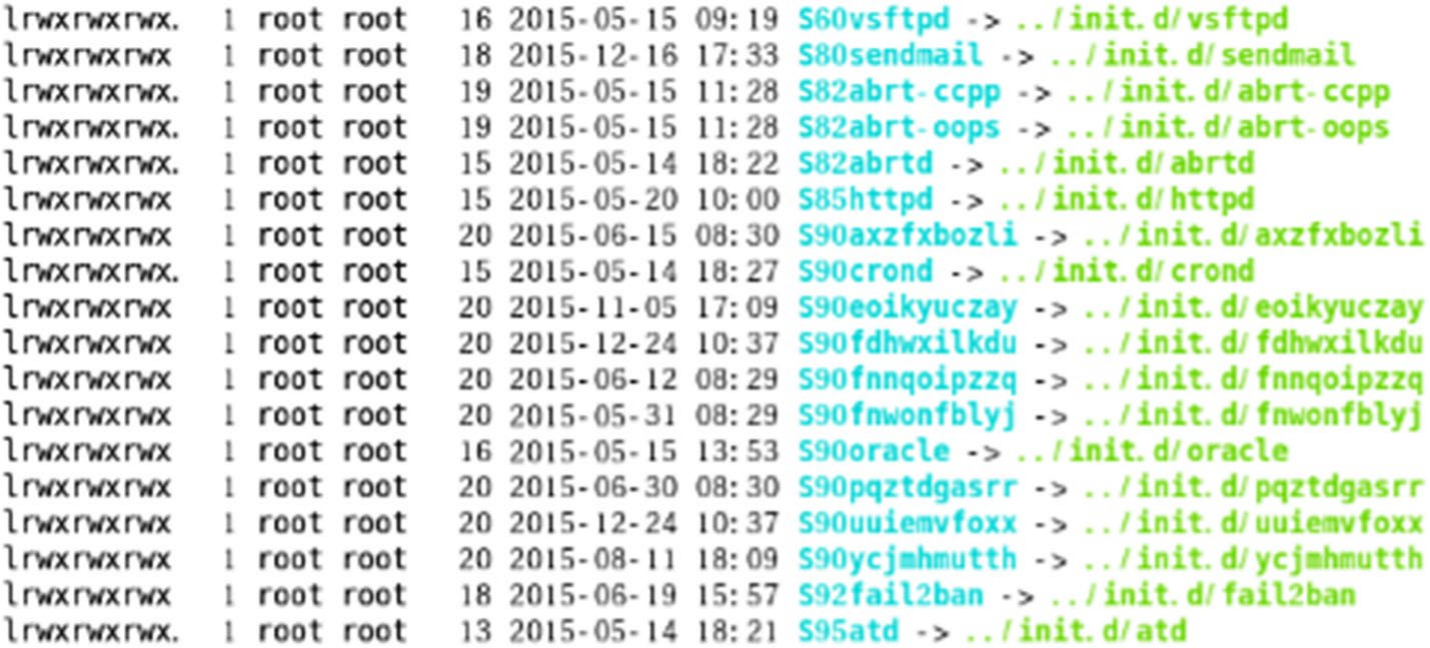
Installation of XOR DDoS malware

Recently, there has been an increase in attackers gaining the administrative rights of Linux-based systems by exploiting web service vulnerabilities such as SQL Injection, OpenSSL, and file uploads. The attackers access the C&C server or malware distribution site with their administrative status, and execute the wget or curl command in order to perform a forced installation of the downloaded malware. Figure 1 shows the installation of the malware program in the/etc./rc.3d and/etc./rc5.d directories after a reboot of a system infected with malware. The installed malware communicates with the C&C server and prepares to launch attacks (Sakib and Huang 2016; Park and Lee 2001).

Table 1 is an example of a SYN flooding payload caused by an IP-spoofed DDoS attack in an actual information service environment.

**Table 1:** Capturing network traffic by XOR DDoS

06:05:24.260515 IP x.x.x.x.7318 > y.y.y.y.80: Flags [S], seq 479609867:479610763,win65535,length896	
06:05:24.260540 IP x.x.x.x.2104 > y.y.y.y.80: Flags [S], seq 137948748:137949644,win65535,length896	
06:05:24.260560 IP x.x.x.x.58852 > y.y.y.y.80: Flags [S], seq 3856952941:3856953837,win65535,length 896	
···	
06:05:24.260574 IP x.x.x.x.4375 > y.y.y.y.80: Flags [S], seq 286734425:286735321,win65535,length896	
06:05:24.260583 IP x.x.x.x.62129 > y.y.y.y.80: Flags [SE], seq 4071711351:4071712247,win65535,length896	

**Fig. 2:**
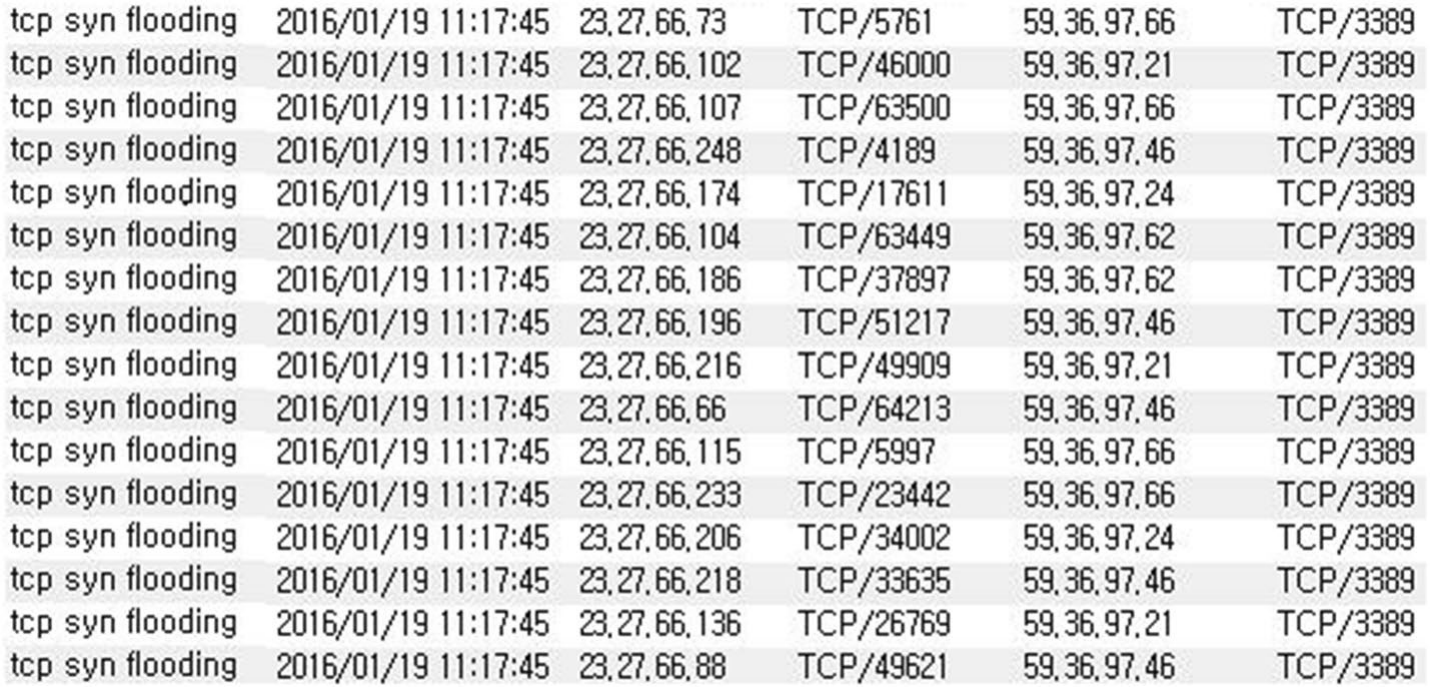
DDoS attacks detected by intrusion detection system

The SYN flooding attacks are launched by the infected system on an external, unspecified target and IP spoofing makes it more difficult to detect the source of the infection. If the infected system behind the attacks cannot be efficiently identified, the bandwidth of the internal network becomes consumed by DDoS attacks, resulting in a disruption of network services (Liu and Bi 2015; Freiling et al. 2005). Figure 2 shows the detection of DDoS attacks generated by a system infected with malware.

**Fig. 3:**
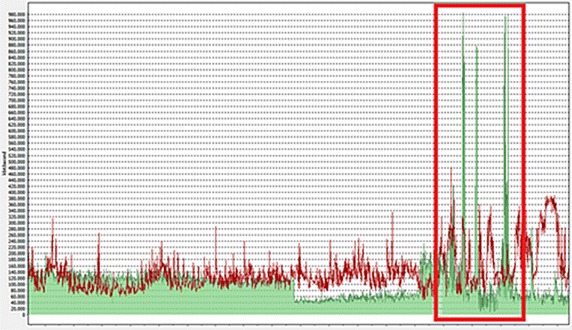
Monitoring of network traffic by XOR DDoS attack

Figure 3 shows the monitoring of the Network Management System (NMS) when infected by IP-spoofed DDoS malware. The screen shows the DMZ network, which provides services with an average traffic of 600 Mbps. As shown, the infected system generated DDoS traffic of 2 Gbps. The unexpected increase in traffic, exceeding the 1 Gbps limit, caused the failure of the entire network.

Ultimately, the most effective way of ensuring network availability is the rapid detection and response to systems that generate huge volumes of traffic after being infected with IP-spoofed DDoS malware. This is also related to the reliability of information services.

One method of preventing malware infection is to install vaccine programs for all Linux-based systems, but this is not the best solution for companies. Installing vaccines on all Linux systems is not only expensive, but also requires the software license to be renewed each year.

**Fig. 4:**
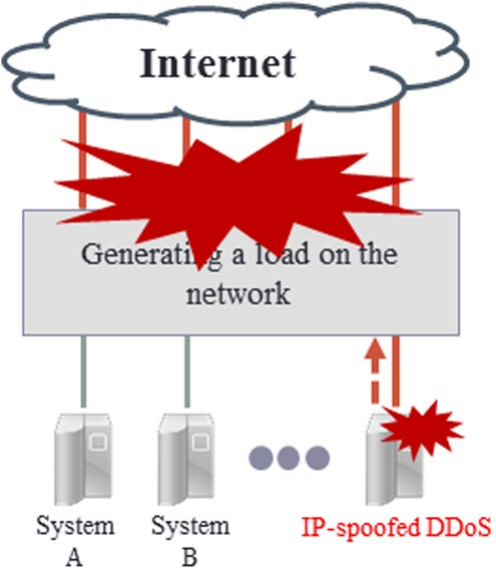
DDoS detection based on network

Another method is to install anti-bot programs to detect malicious bots in the local system. However, anti-bot programs may be impossible to install depending on hardware performance and additional licenses have to be purchased if new hosts are added. As illustrated in Fig. 4, it is important to consider a network-based approach to malware detection in order to overcome the aforementioned issues.

**Fig. 5:**
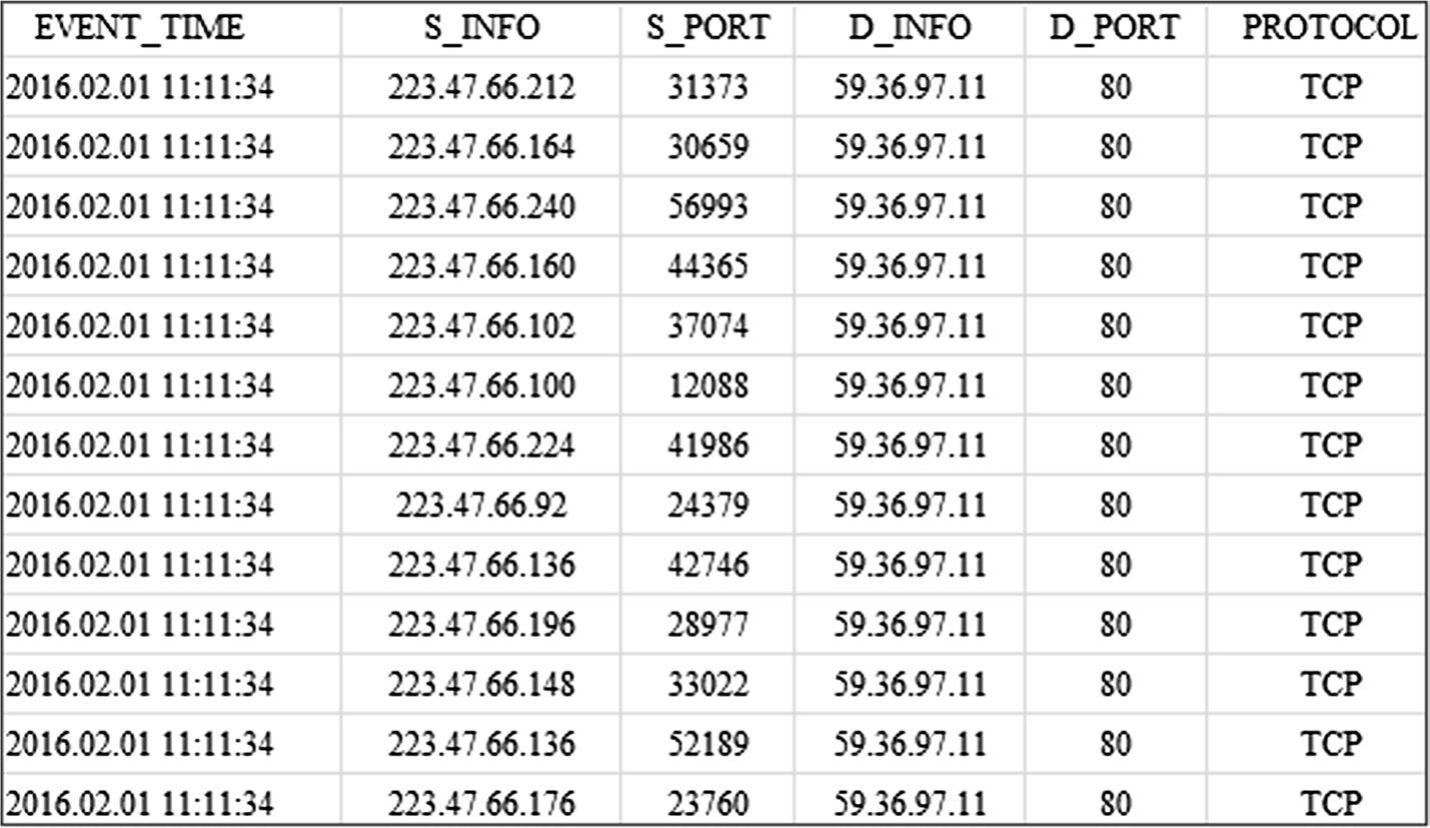
IP address and port number of IP-Spoofed DDoS attacks

By analyzing an actual system infected with IP-spoofed DDoS malware, the following features were identified. First, massive volumes of SYN flooding attacks were generated by creating more than two million SYN packets per minute. Second, IP spoofing was performed in order to conceal the source of the attacks. The list of attacked systems was downloaded from the C&C server and periodically updated for the next series of attacks. Figure 5 shows the IP address and port number of IP-spoofed DDoS attacks.

This study proposes a method that can detect network-based IP-spoofed DDoS attacks and efficiently identify malware-infected systems.

## System overview

The DMF algorithm analyzes the feature information of the traffic header in order to detect IP-spoofed DDoS malware. The three phases are extraction, analysis, and detection. In the first phase, the attack features of the IP-spoofed DDoS malware are analyzed after categorizing the internal network by service area and collecting real-time network traffic. Based on the Layer 3 switch, network services are categorized into user bandwidth, server farm bandwidth, and branch office. Traffic collection is configured in port mirroring and a DMF table is created by extracting feature information, including the IP address and MAC address of the Ethernet headers, the current time, and the time interval. In Table 2, the columns specify the attribute information of a DMF table.

**Table 2:** Attribute information of a DMF table

Attribute	Type	Feature data	Description
SN	VARCHAR(20)	S00001	Sequence number to traffic
SGN	VARCHAR(20)	G00001	Same group number with the same destination IP
Attribute Info.			
SRCIP	VARCHAR(15)	58.203.201.36	Source IP
SRCPORT	VARCHAR(6)	5312	Source Port
DSTIP	VARCHAR(15)	211.106.66.102	Destination IP
DSTPORT	VARCHAR(6)	80	Destination Port
MAC	VARCHAR(17)	D0-27-88-47-15-4B	Media access control (MAC)
PT	VARCHAR(8)	TCP	Protocol
AT	DATE	2016.08.12 07:05:28	Destination IP connection time
TIN	FLOAT	2 s	Interval from previous traffic
CND	INTEGER	28 hit	Destination IP connection hits
RATD	FLOAT	136 s	Interval from previous traffic connected to destination IP
CNTI	INTEGER	12 hit	Hits to the destination IP over 3 min
STATE	VARCHAR(20)	Abnormal	Traffic anomaly status

**Fig. 6:**
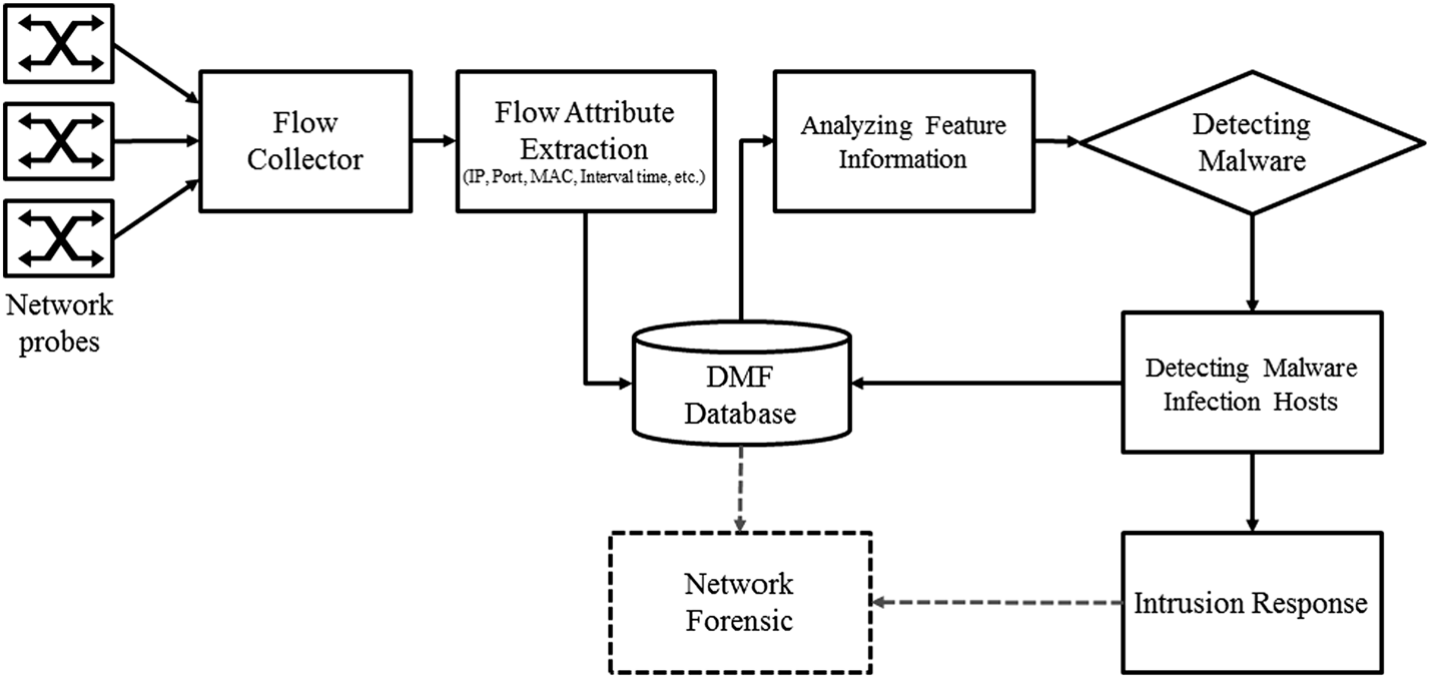
Process for detection of IP-spoofed DDoS malware

In the second phase, the proposed detection algorithm is applied and statistical features are analyzed in order to detect IP-spoofed DDoS malware. The extracted IP addresses and MAC addresses from Ethernet headers of real-time traffic are compared with the attribute information provided in the DMF table to determine if IP-spoofed DDoS malware infection has occurred. Figure 6 shows the process for detection of IP-spoofed DDoS malware.

The DMF table consists of the attribute information of traffic headers. The time interval is calculated if there is a match between the IP addresses and MAC addresses of real-time traffic with the property values in the DMF DB. Time-interval values are used to check for IP-spoofed DDoS malware infection.

Instead of referring to traffic payload, the proposed algorithm relies on the feature information of the IP-spoofed DDoS malware for malware detection.

## System model

The purpose of the experiment is to detect malware-infected systems after detecting IP-spoofed DDoS based on an analysis of real-time traffic. This section examines the system model for the proposed method.

### Gathering on the network traffic

**Fig. 7:**
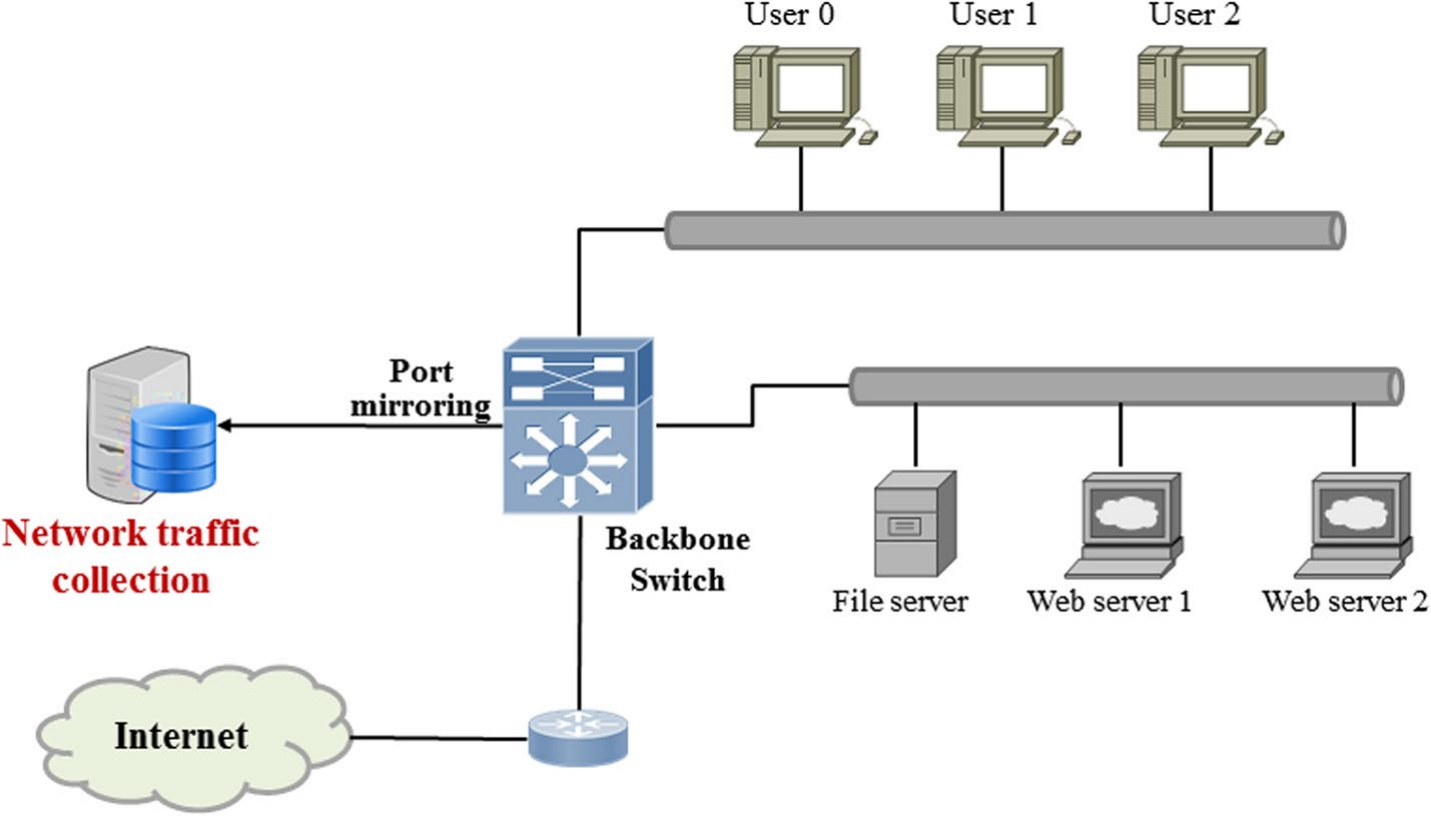
Collection and analysis of network packets

To implement the DMF algorithm, port mirroring is configured at the Layer 3 switch. The reason for the port mirroring configuration is to detect malware infection based on an analysis of traffic routed through the network equipment and to identify infected systems. Figure 7 shows the schematic diagram for real-time network traffic collection.

### Extraction on the feature information

**Fig. 8:**
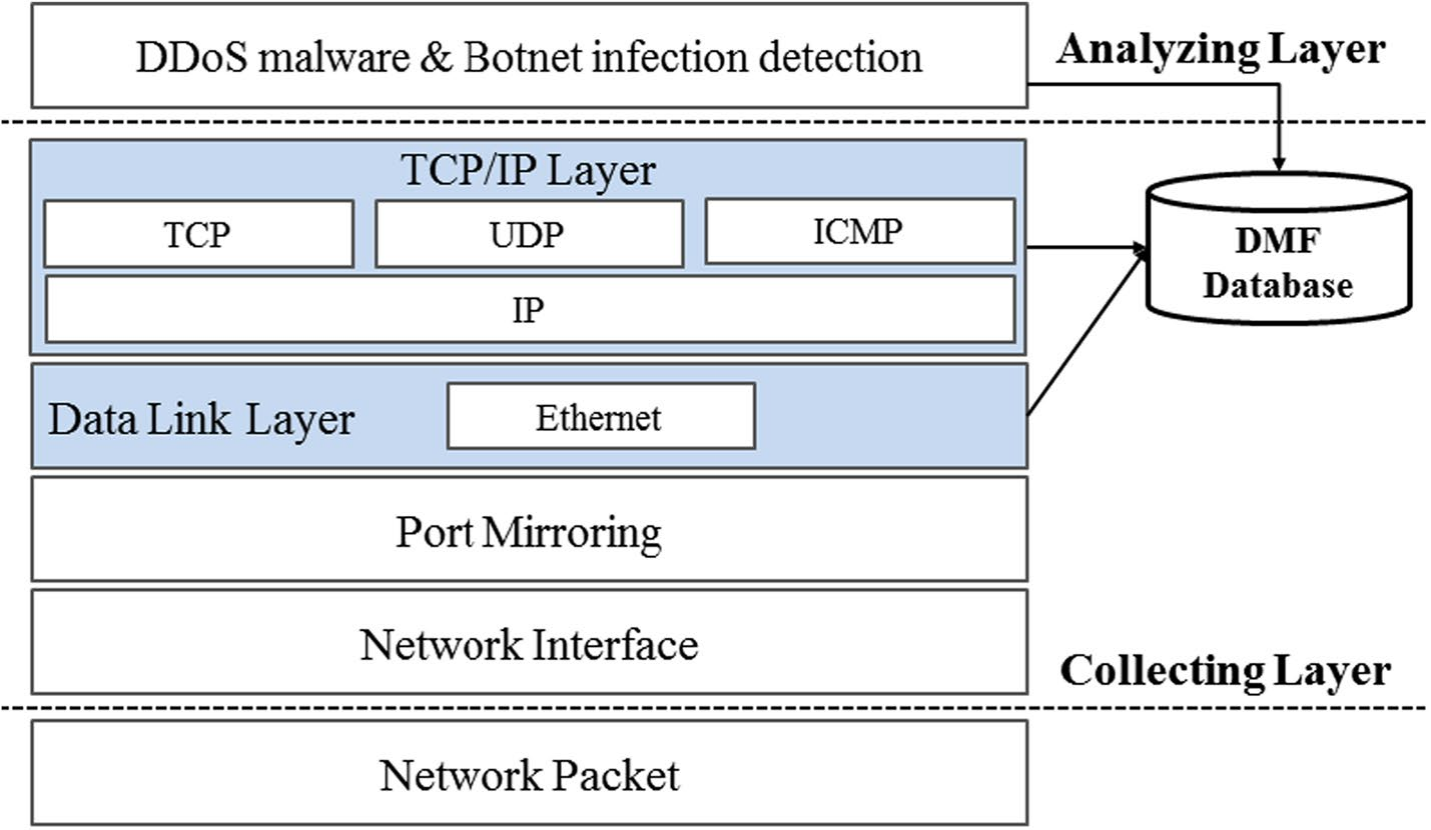
Protocol stack for extraction of feature information

As shown in Fig. 7, the attribute information by TCP/IP and Ethernet header are extracted from the collected traffic and used as basic data in generating the DMF DB. Figure 8 shows the protocol stack used in extracting the feature information from real-time traffic.

**Fig. 9:**
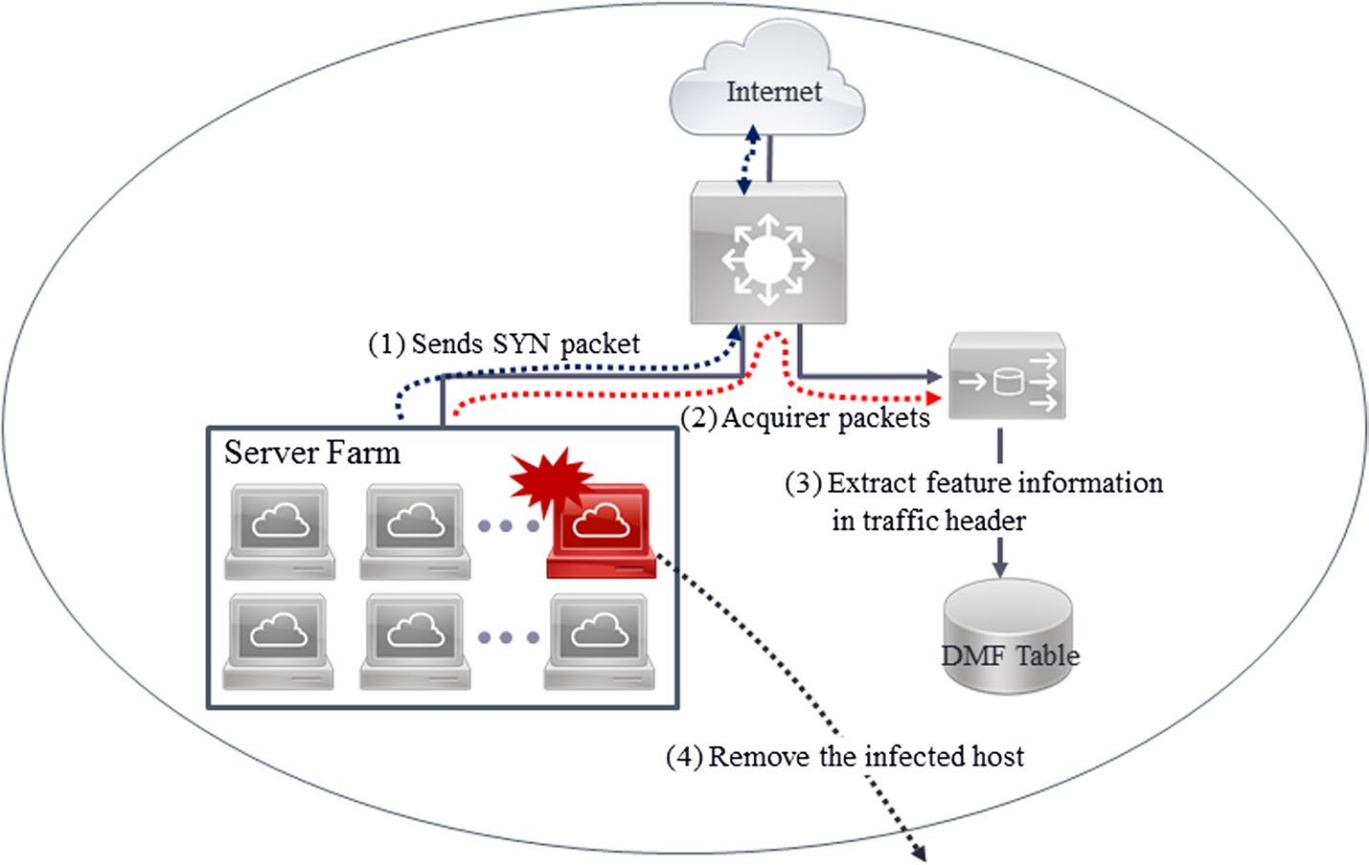
Extraction of feature information

As shown in Fig. 8, the headers of traffic flowing out of the internal network are used to extract feature information. Figure 9 shows the process of collecting SYN packets sent by the server farm and extracting the feature information from the collected traffic.

### Creation of the DMF table

The DMF table is used to detect DDoS attacks in real-time traffic and contains feature values that are useful in identifying malware-infected systems. The procedures for generating the DMF table are as follows:

- The SN field of the DMF table represents the traffic sequence and the SGN field means the number of the group connecting to the same destination IP.
- The traffic header IP address and MAC address is extracted and stored in the SRCIP, DSTIP, and MAC fields for the DMF table. The communication protocol between the two hosts and the access time are also stored.
- The TIN field represents the interval between previous traffic and current traffic.
- The CND field represents the connection hits to the destination IP and the RATD field represents the reconnect interval of the destination IP.
- The STATE field is categorized into two types. If the TIN field value and RATD value are zero and the CNTI field divided by 180 s is ≤1 then the field value is set as “abnormal”. All other instances are set as “normal”. Table 3 shows the algorithm for creating the DMF table.

**Table 3:**
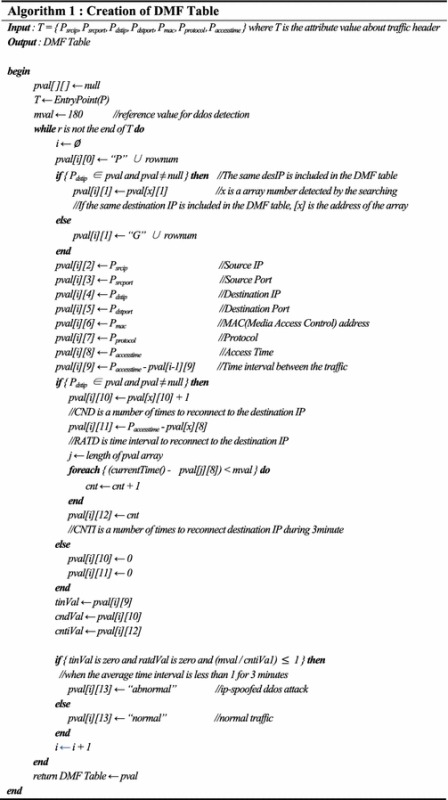
Creation of DMF table

**Fig. 10:**
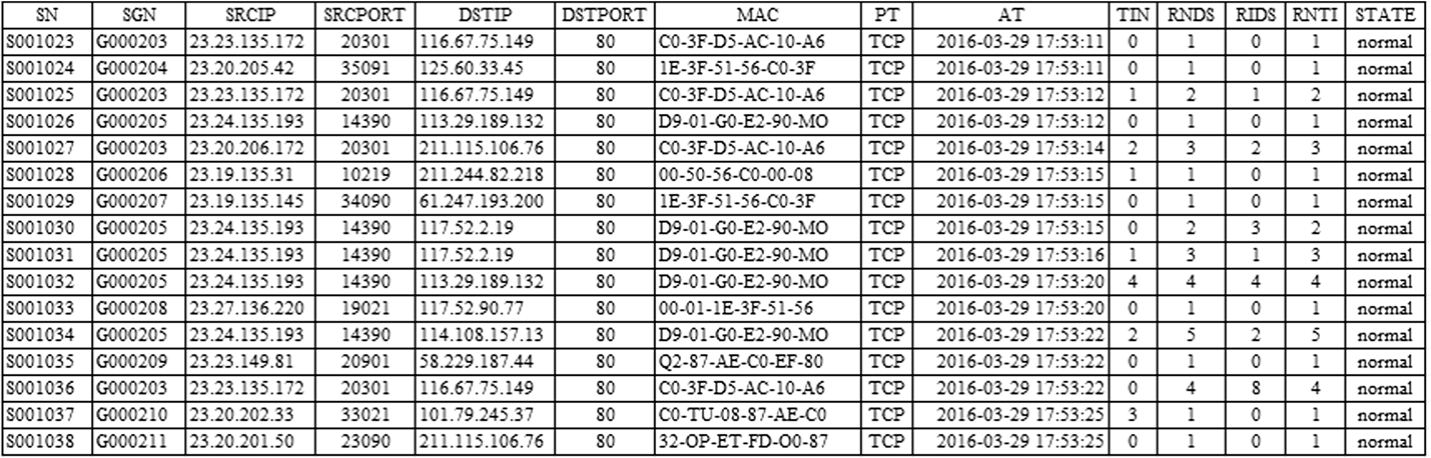
Feature information of collected traffic

Figure 10 shows an example of the data stored in the DMF table.

### Analysis of feature information

**Fig. 11:**
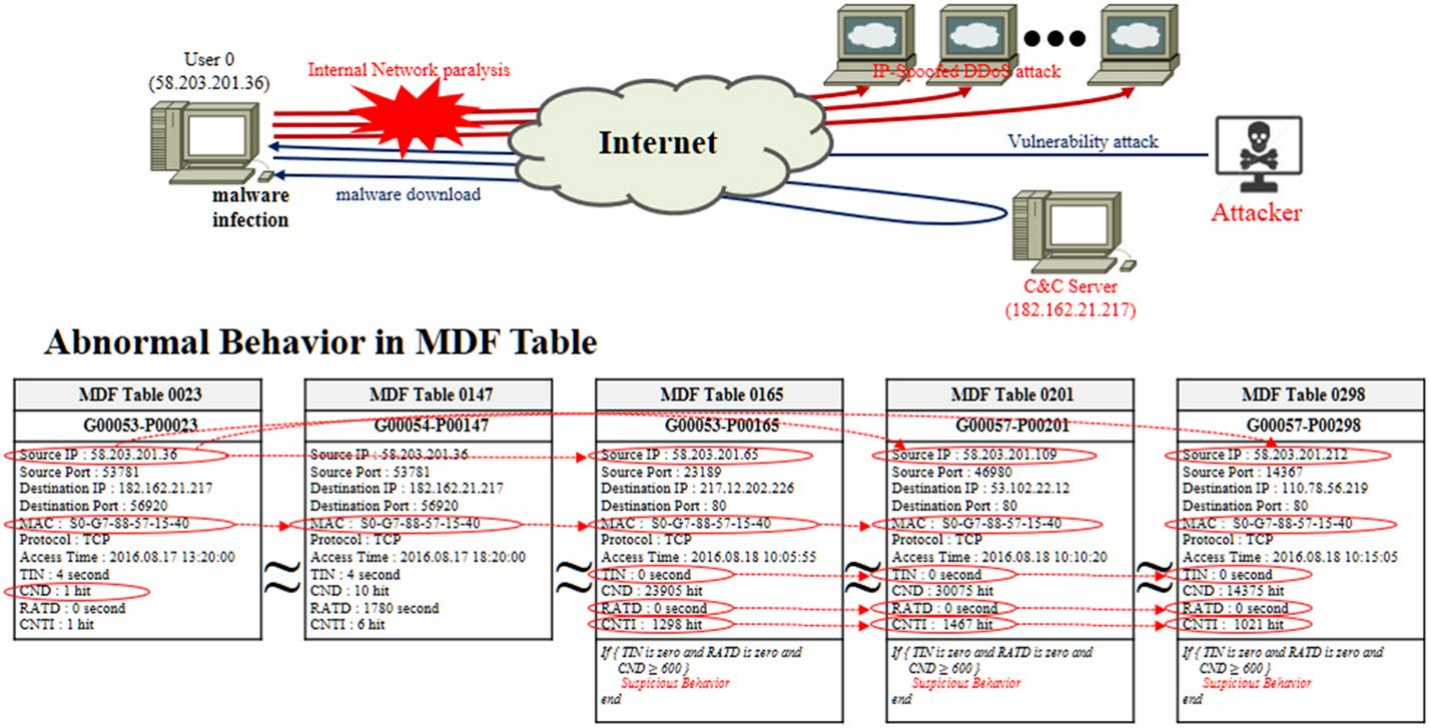
Analysis of feature information using DMF table

Malware-infected hosts receive attack commands from the C&C server or leaks important data to the C&C server. Also, they receive DDoS attack commands from the C&C server or generate large scale IP-spoofed DDoS attack traffic at a specified time. According to the performance of the host and the network bandwidth, the DDoS traffic generated at this time can cripple the internal network and makes it extremely difficult to detect IP-spoofed malware-infected hosts. The proposed method to detect the DDoS attack is to use the DMF table in order to analyze the traffic generation time and generation frequency, thereby determining if it is IP-spoofed by using the IP address and MAC address. To detect malware-infected hosts, the connection times to the MAC address and destination IP address and connection frequency are used. Figure 11 shows a DMF table with the analysis results of the attribute information of the network traffic.

### Detection of IP-spoofed DDoS attack

In order to detect IP-spoofed DDoS attacks, attribute information collected from the core network is analyzed. Attribute information is stored in the DMF table and, as seen in Fig. 11, the attribute’s relative information is used to detect attacks. Table 4 shows the algorithm to detect IP-spoofed DDoS attacks.

**Table 4:**
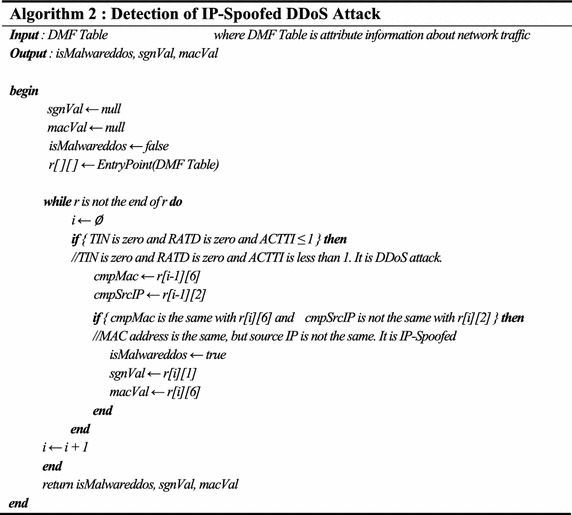
Detection of IP-spoofed DDoS attack

**Fig. 12:**
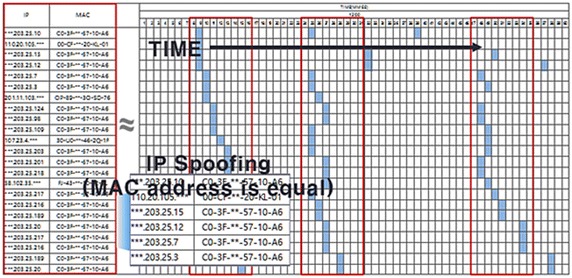
Timeline of an IP-spoofed DDoS attack in a malware-infected System

Figure 12 shows the timeline of traffic information generated on a network when a malware-infected host executes an IP-spoofed DDoS attack.

As shown in Fig. 12, analysis results of the attributes of IP-spoofed DDoS malware showed that it was periodically communicating with the C&C server and the attack target was downloaded from the C&C server and updated. The IP-spoofed DDoS attack was powerful enough to cause an outage to the core network in a gigabit Ethernet environment.

### Finding of a system infected by IP-spoofed DDoS malware

Upon detecting the IP-spoofed DDoS attack with the proposed methodology, it is necessary to find the malware-infected host and eliminate the cause. Because of IP address spoofing, it is impossible to block a particular IP address in the firewall, and even if we were to block a range of IP addresses, the internal network would either come to a halt or have extreme delays as a result of the large volume traffic generated by the malware. Therefore, it is necessary to quickly quarantine the host that is generating the DDoS attacks to an edge network.

IP-spoofed DDoS attacks create tampered IP address packets on a host infected by malicious code and utilizes the maximum performance of the host to generate large volume traffic. According to the DDoS attack, network resources are consumed and because the web services and intranet services are not executed normally, resources are depleted as time goes by. Table 5 shows an algorithm to effectively detect malware-infected hosts.

**Table 5:**
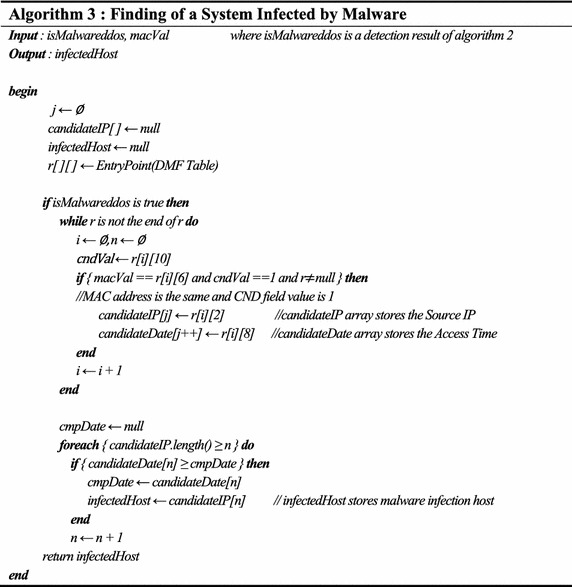
Finding of a system infected by malware

### Intrusion response on infected system

**Fig. 13:**
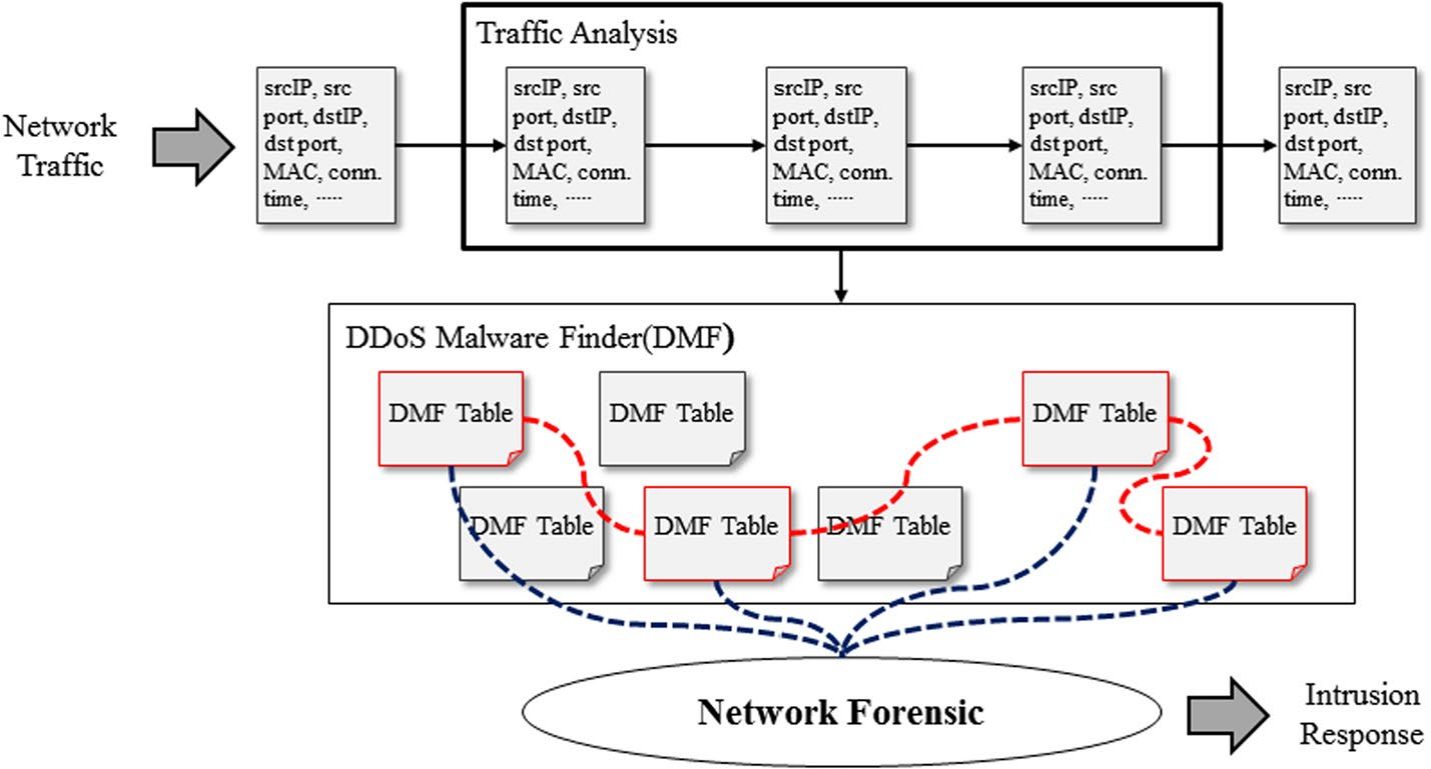
Network forensic using DMF table

When a DDoS attack manifests in the internal network, it is necessary to detect the intruding system and respond before it consumes all of the internal network’s bandwidth. Also, it is necessary to understand the intrusion cause and secure the audit trail in order to eliminate any weak spots. The proposed method is for network forensics to understand the traffic flow and to use the attribute values of the DMF table to analyze the malware-infected system and its attack tendencies. Figure 13 shows the network forensics execution plan using the DMF table.

## Evaluation

**Fig. 14:**
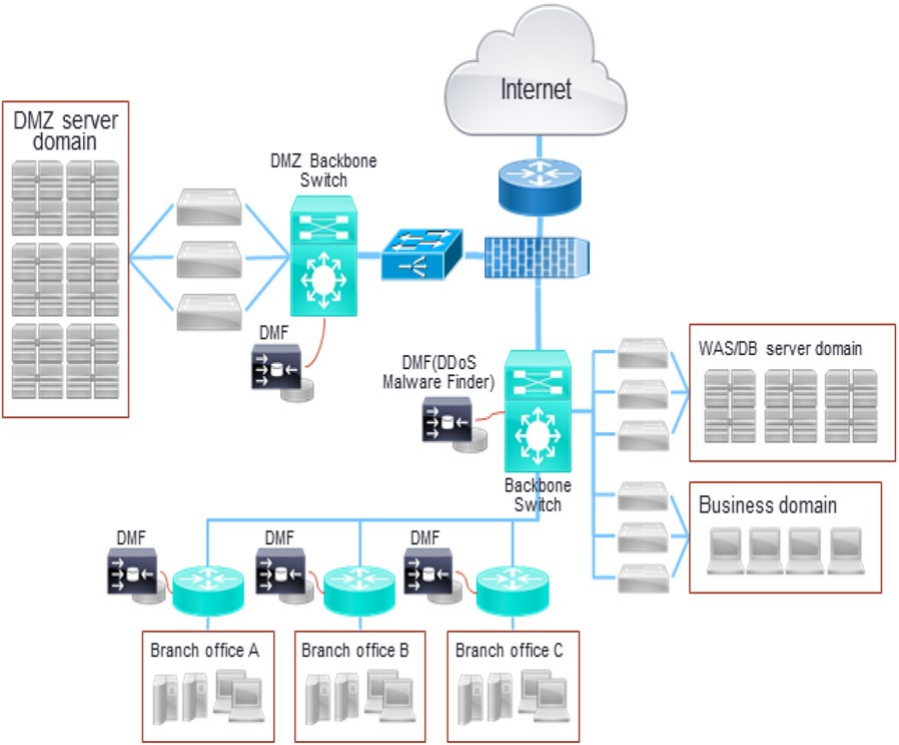
Diagram of experiment environment

The testbed environment is organized as shown in Fig. 14 in order to evaluate the efficiency of the proposed algorithm, detect IP-spoofed DDoS attacks, and identify malware-infected systems. This section describes the differences between the proposed method and existing technology and evaluates the efficiency of detecting local hosts infected with IP-spoofed DDoS malware.

 The test environment for the assessment of the proposed algorithm was a Gigabit Ethernet network environment with IP-spoofed DDoS malware installed in the server farm of the DMZ network. For the installation of the proposed algorithm, the system configuration included an Intel i7 CPU with eight cores, 16 GB of RAM, and 2Tbyte of HDD. The Java programming language was used to program the prototype in order to verify the performance of the proposed algorithm.

### Comparison of the proposed method and the intrusion detection system

When malware is installed via zero-day vulnerability or drive by download, the security system can no longer guarantee the stability of the internal network. Host-based anti-malware solutions could possibly be effective, but applying it to large-scale networks poses administrative and technical difficulties. Therefore, the proposed algorithm can detect network based IP-spoofed DDoS attacks and effectively detect malware-infected hosts. Table 6 compares the performance of the proposed algorithm and the IDS.

**Table 6:** The performance comparison of the proposed algorithm and IDS

Performance	Proposed algorithm	Intrusion detection system (IDS)
Detection of DDoS attack	Support	Support
Detection of IP-spoofed DDoS attack	Support	Not supported
Detection of system infected by malware	Support	Not supported

Results of the verification of the proposed algorithm and the IDS on a testing environment showed that both were able to adequately detect DDoS attacks; however, the IDS could not detect IP-spoofed DDoS attacks and malware-infected hosts.

**Fig. 15:**
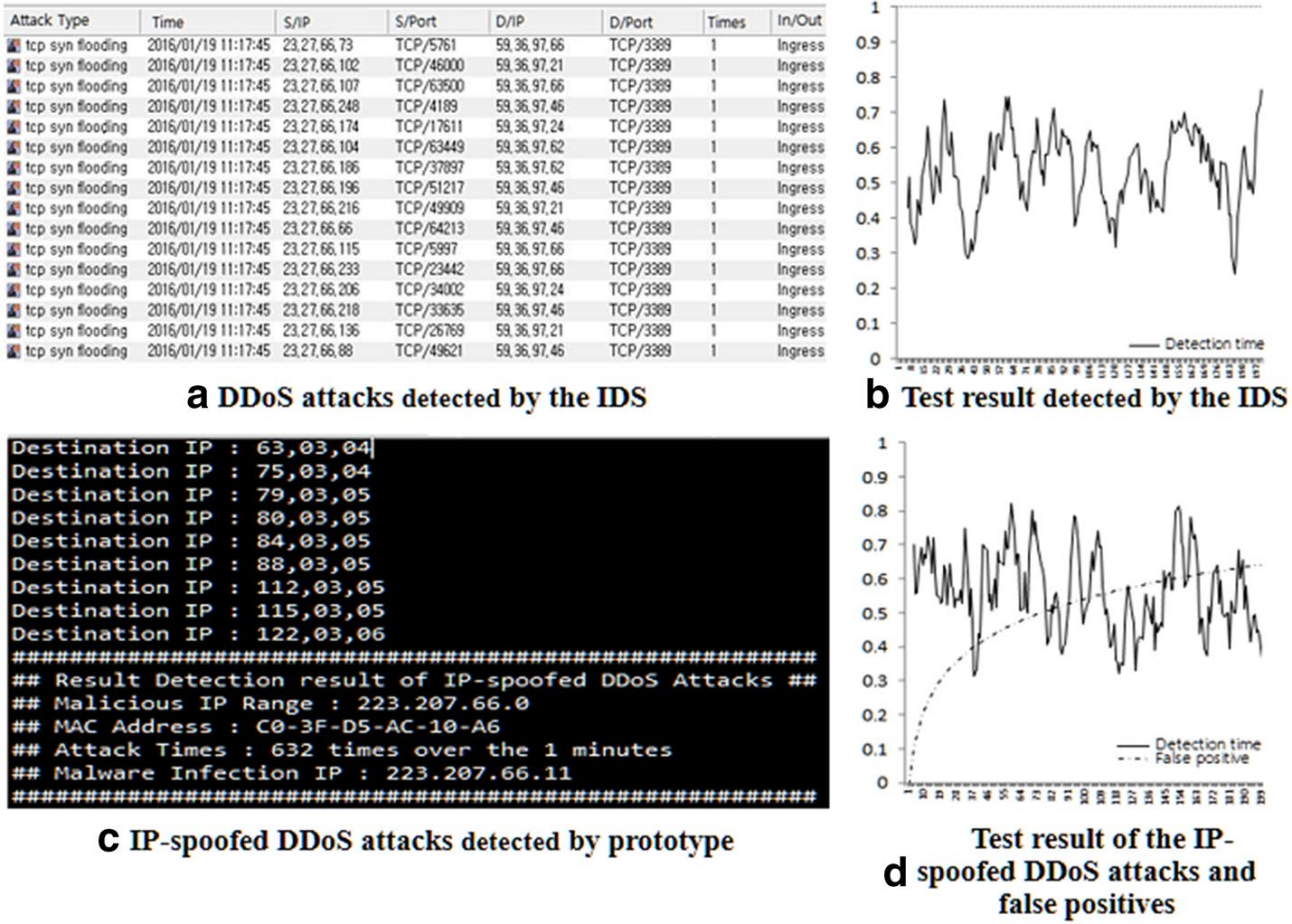
A comparative analysis of the proposed algorithm and IDS

Figure 15a is the result of DDoS attacks detected by the IDS, and Fig. 15b shows the time that DDoS attacks were detected. Figure 15c shows the results of IP-spoofed DDoS attacks detected by prototype implemented with the proposed algorithm. Figure 15d shows the detection times of the IP-spoofed DDoS attacks and false positives in the test environment.

### Detection time of IP-spoofed DDoS malware

**Fig. 16:**
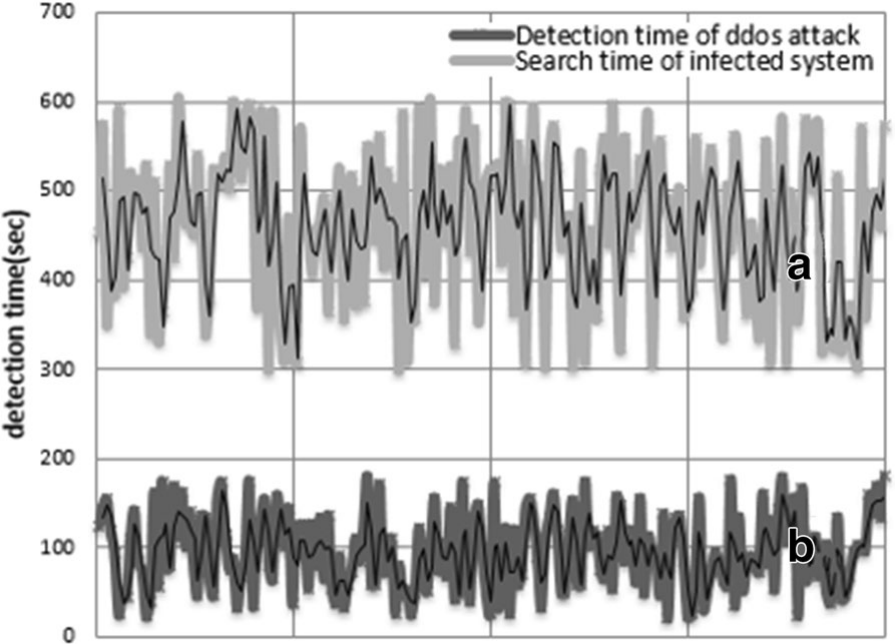
Test result (detection time of infected system by malware)

The purpose of this study is to detect and rapidly respond to malware before network resources are depleted when massive volumes of DDoS attacks are launched by a system infected with IP-spoofed DDoS malware. Tests were performed using the proposed method, and the detection times of DDoS attacks are as shown in Fig. 16.

Figure 16 shows the detection time for IP-spoofed DDoS attacks and malware-infected systems. Graph A represents the time at which IP-spoofed DDoS attacks occur due to the local host being infected with malware, while Graph B is the discovery time of when the local host has been infected with IP-spoofed DDoS malware.

**Fig. 17:**
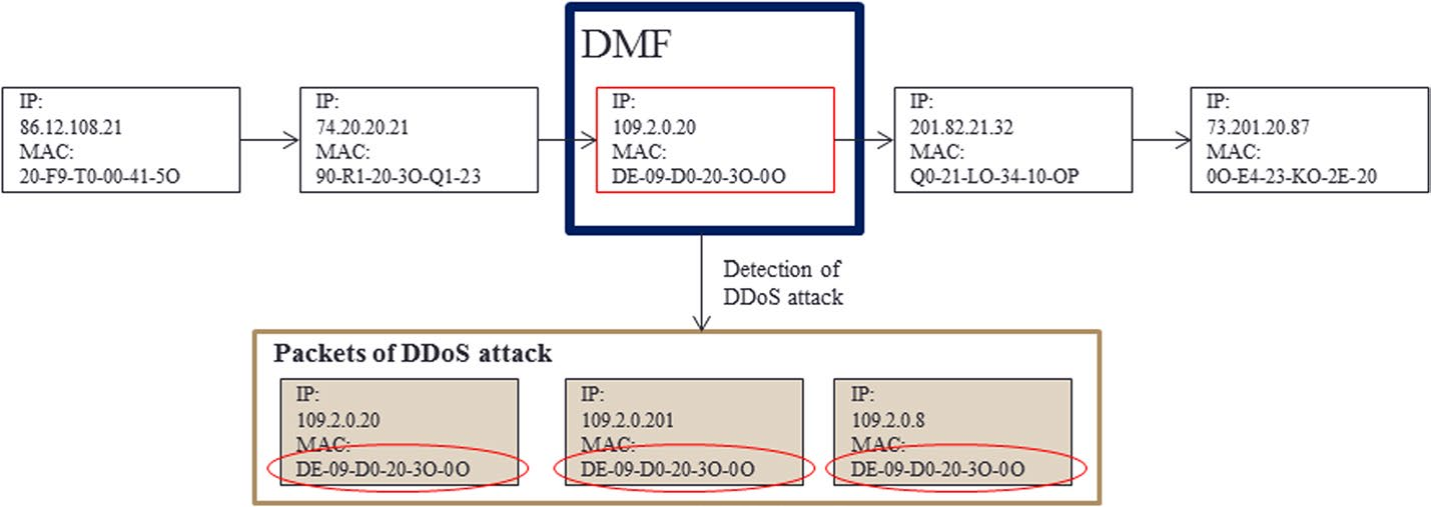
Detection of IP-spoofed DDoS attacks using DMF algorithm

The average detection time under the proposed algorithm for IP-spoofed DDoS attacks in the local host was 98 s; the longest time was 179 s. Figure 17 shows the detection mechanism for DDoS attacks. The proposed algorithm analyzes the headers of real-time traffic to detect IP-spoofed DDoS attacks.

**Fig. 18:**
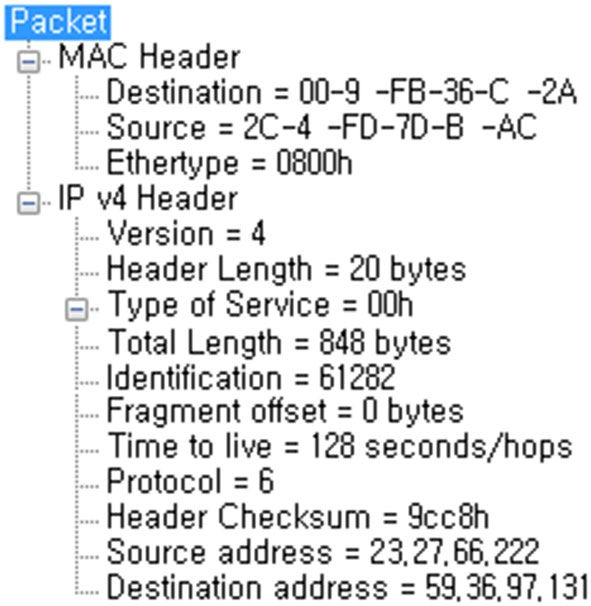
Packet information collected by DMF

Figure 18 shows the packet information collected by the DMF. The average time taken to detect systems infected with IP-spoofed DDoS malware was 444 s; the longest time was 600 s.

### Accuracy

To assess the accuracy of detecting malware-infected systems using the proposed method, tests were carried out under the test environment in Fig. 14. Accuracy tests were performed under the following conditions:

- Installation of malware in the test server and launch of IP-spoofed DDoS attacks.
- Analysis of attack features and false positives after varying the IP address of the test server.
- Analysis of attack features and false positives after replacing the server hardware.

The accuracy of detecting malware-infected systems was calculated as follows:

$$Accuracy\,(\% ) = 1 - \left( {\frac{false\;positive}{test\;number}} \right) \times 100$$

The experimental results are presented in Table 7.

**Table 7:** Accuracy of experiment result

Error ratio	Accuracy (%)	Explanation
12:1000	98.8	TP rate = 0.988, FP = 12
8:600	98.7	TP rate = 0.987, FP = 8
2:400	99.5	TP rate = 0.995, FP = 2
1:100	99.0	TP rate = 0.99, FP = 1
0:50	100	TP rate = 1, FP = 0

The accuracy of detecting systems infected with IP-spoofed DDoS malware was assessed under the test environment described above, and averages were obtained from 1000 runs. The detection accuracy was approximately 98% and 12 false positives occurred during the experiment. False positives were detected when there was an error with the network configuration or when ARP spoofing occurred.

### Effectiveness

Past research has focused on the detection of outbound-to-inbound DDoS attacks or malware-infected hosts in internal network. However, various challenges have yet to be addressed for detecting and responding to inbound-to-outbound DDoS attacks launched by malware-infected local hosts. If DDoS attacks originating from local hosts cannot be effectively resolved, normal network services become difficult due to bandwidth exhaustion. This experiment found that the malware-infected system generated more than one million SYN flooding packets per minute and the network traffic was approximately 64 GB.

**Fig. 19:**
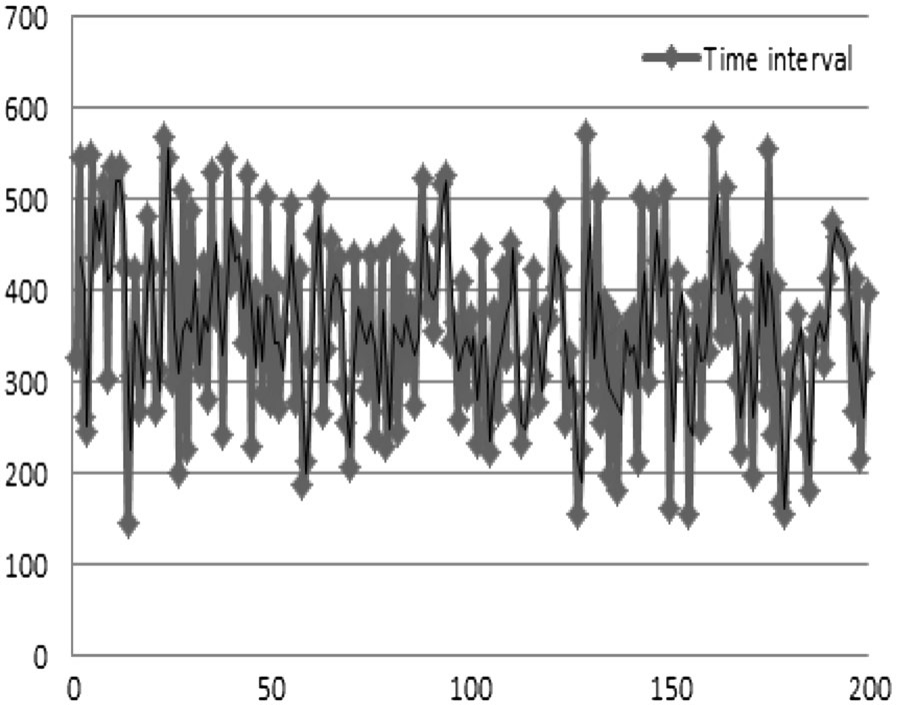
Time interval to detect the system infected by malware

Agents do not have to be separately installed in order to detect malware-infected systems using the proposed method, and widespread detection is possible for malware-infected systems running on networks. As shown in Fig. 19, the average time taken to detect a system infected with IP-spoofed DDoS malware was 542 s.

$$\begin{aligned} {\text{Mean}}\;{\text{time}}\;{\text{for}}\;{\text{intrusion}}\;{\text{response}}\,({\text{MTIR}}) & = {\text{detection}}\;{\text{time}}\;{\text{of}}\;{\text{IP}}\;{\text{spoofing}}\;{\text{DDoS}}\;{\text{attack}} \\ & \quad + {\text{detection}}\;{\text{time}}\;{\text{of}}\;{\text{infected}}\;{\text{system}} \\ \end{aligned}$$

### False positives

This proposed method enables the effective detection of IP-spoofed DDoS attacks and malware-infected systems. False positives were detected in two cases. In the first case, errors in network configuration after server replacement caused anomalous traffic. In the second case, changes in the MAC address under ARP spoofing resulted in anomalous traffic. In actual information system environments, errors in network configuration lead to problems in communication, thereby allowing them to be identified before operating information services. The detection of ARP spoofing indicates an intrusion of the internal network and adequate measures must be taken to remove the malware.

## Conclusion

A key factor in enhancing corporate reliability is the stability of information services provided through the Internet. While various security solutions are available today, DDoS attacks still pose significant threats to information service providers and security administrators.

When a system infected with IP-spoofed DDoS malware launches massive volumes of DDoS traffic in the internal network, a fundamental solution is to detect and remove the malware-infected system. This is because flooding packets generated by the local host affect network communications using the Interior Gateway Protocol (IGP) as well as the Exterior Gateway Protocol (EGP). A real-world example is the 6-h halting of network services by an Internet service provider under an IP-spoofed DDoS attack despite being equipped with various security solutions, such as DDoS countering equipment and an intrusion prevention system (Strayer et al. 2006; Stone-Gross et al. 2009). If DDoS attacks are not detected in advance and responded to, there is likely to be a negative impact on the reliability of information services provided by such companies.

Using the algorithm proposed in this paper, it is possible to effectively detect IP-spoofed DDoS attacks and rapidly respond to malware-infected systems. The proposed algorithm derives patterns from features of IP-spoofed DDoS malware and matches information in the headers of real-time traffic with DMF table property values to detect IP-spoofed DDoS attacks and malware-infected systems. The efficiency of the proposed algorithm was demonstrated through various experiments and its applicability to actual operating environments was verified under a testbed environment.
